# Extracellular Vesicles as Therapeutic Strategy for Ischemic Stroke

**DOI:** 10.1111/jnc.70311

**Published:** 2025-11-25

**Authors:** Elisama Araújo da Silva, Júlio César Queiroz Figueiredo, Erik Aranha Rossi, Rachel Santana Cunha, Flávia Santos Sanches, Adne Vitória Rocha de Lima, Erick Correia Loiola, Zaquer Suzana Munhoz Costa‐Ferro, Bruno Solano de Freitas Souza

**Affiliations:** ^1^ Gonçalo Moniz Institute Oswaldo Cruz Foundation (FIOCRUZ) Salvador Brazil; ^2^ Center for Biotechnology and Cell Therapy São Rafael Hospital Salvador Brazil; ^3^ D'Or Institute for Research and Education (IDOR) Salvador Brazil; ^4^ Pioneer Science Initiative D'Or Institute for Research and Education (IDOR) Rio de Janeiro Brazil

**Keywords:** extracellular vesicles, immunomodulation, ischemic stroke, neural regeneration, therapeutic strategies

## Abstract

Ischemic stroke remains one of the leading causes of death and long‐term disability worldwide, with current treatments limited by narrow therapeutic windows and the risk of hemorrhagic transformation. In this context, extracellular vesicles (EVs) have emerged as a promising cell‐free therapeutic strategy due to their ability to modulate inflammation and support neuroregeneration. This review explores recent advances in the application of EVs in ischemic stroke therapy, highlighting their mechanisms of action, including the delivery of neuroprotective molecules such as microRNAs and proteins that promote angiogenesis, neurogenesis, and anti‐apoptotic pathways. We summarize findings from preclinical models demonstrating the regenerative potential of EVs derived from mesenchymal stem cells, microglia, neural progenitor cells, and other cell types, as well as advances in bioengineered EVs for targeted delivery. Despite encouraging results, the clinical translation of EV‐based therapies faces challenges, including large‐scale production, content variability, and targeted delivery efficiency. Future efforts should focus on optimizing EV characterization and manufacturing processes to ensure therapeutic consistency and safety.

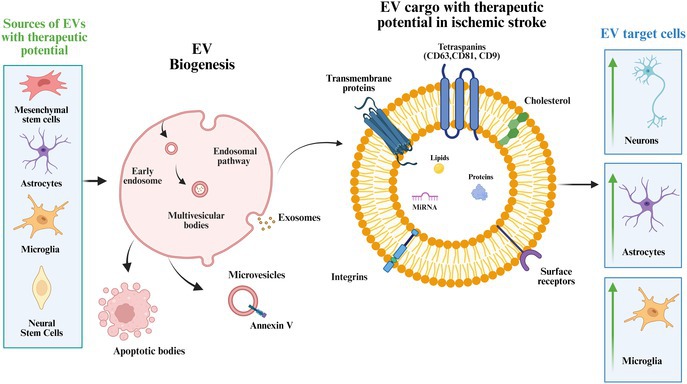

## Graphical abstract

Ischemic stroke causes inflammatory and degenerative cascades. We review how extracellular vesicles from stem and brain cells modulate these pathways and promote repair, summarize key bioactive cargo, and define the critical steps for clinical translation (standardized manufacturing, dosing, potency, safety). Extracellular vesicles (EVs) may complement current therapies by enabling post‐acute neurorestoration.

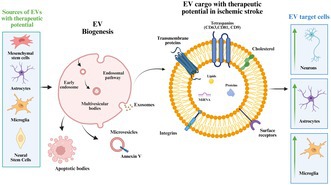

**Abbreviations**
ASCs: adipose‐derived stem cells
ATP: adenosine triphosphate
BBB: blood–brain barrier
BDNF: brain‐derived neurotrophic factor
BMSC‐sEVs: bone marrow mesenchymal stem cells
Ca^2+^/CaMK II: calcium/calmodulin‐dependent protein kinase II
CBF: cerebral blood flow
EPC: endothelial progenitor cell
EVs: extracellular vesicles
GMP: Good Manufacturing Practice
GPx: glutathione peroxidase
HSHS: Houshiheisan
HSP27: heat shock protein 27
HUCPVCs: human umbilical cord perivascular cells
IC: stereotactic intracerebral infusion
ICAM‐1: intercellular adhesion molecule
ICV: intracerebroventricular injection
IL‐1: Interleukin 1
IL‐10: Interleukin 10
IL‐6: Interleukin 6
IN: intranasal injection
IP: intraperitoneal injection
iPSCs: induced pluripotent stem cells
ISEV: International Society for Extracellular Vesicles
IV: intravenous injection
KMO: kynurenine 3‐monooxygenase
MCAO: middle cerebral artery occlusion
MMP‐9: matrix metalloproteinase‐9
MPTP: mitochondrial permeability transition pore
MSC‐EVs: mesenchymal stem cell‐derived extracellular vesicles
MSCs: mesenchymal stem cells
NMDA: N‐methyl‐D‐aspartate receptor
NPC: neural progenitor cell
NPC‐EVs: neural progenitor cell‐derived extracellular vesicles
OGD: oxygen–glucose deprivation
OGD/R: oxygen–glucose deprivation/reperfusion
ROS: reactive oxygen species
rtPA: recombinant tissue plasminogen activator
RVG: rabies virus glycoprotein
RVG29: rabies virus glycoprotein 29
RVG‐sEVs: rabies virus glycoprotein‐functionalized small extracellular vesicles
sEVs: small extracellular vesicles
SOD: superoxide dismutase
TGF‐β1: transforming growth factor beta 1
TNF‐α: tumor necrosis factor alpha
TNK: tenecteplase
TrkB: tropomyosin receptor kinase B
TUNEL: terminal deoxynucleotidyl transferase dUTP Nick End Labeling
VCAM‐1: vascular cell adhesion molecule 1
VEGF: vascular endothelial growth factor

## 1 Introduction

Stroke remains one of the leading causes of disability and mortality worldwide. Despite advances in preventive strategies, both the incidence and prevalence of stroke continue to rise, with a 47% increase in stroke‐related deaths between 1990 and 2021. In 2021 alone, approximately 7 million stroke‐related deaths were reported (Martin et al. 2024). Projections estimate that global stroke cases may increase by 50% by 2050, reaching approximately 21.43 million individuals (Cheng et al. 2024) The long‐term consequences of stroke, in combination with the high costs of pharmacological treatments and rehabilitation, pose a substantial economic burden, with total expenditures in the United States alone nearing US$100 billion (Gerstl et al. 2023).

In recent years, significant advances have been made in the treatment of ischemic stroke, particularly with the introduction of thrombolytic agents. Recombinant tissue plasminogen activator (rtPA) remains the standard of care for acute ischemic stroke and is most effective when administered within 4.5 h of symptom onset, following the exclusion of intracranial hemorrhage (Pedro et al. 2020). More recently, tenecteplase (TNK), a fibrin‐specific thrombolytic agent with higher fibrin affinity and greater resistance to plasminogen activator inhibitors, has emerged as a promising alternative. Clinical studies have demonstrated that TNK is non‐inferior to alteplase in terms of efficacy and exhibits a comparable safety profile (Hagag et al. 2025) Besides that, mechanical thrombectomy, and pharmacological interventions can be applied aiming at restoring cerebral blood flow. Additionally, alternative approaches have been developed for patients who are not eligible for these standard therapies (Lapchak and Zhang 2017). Despite these advancements, several limitations persist, including the narrow therapeutic time window and the potential risk of hemorrhagic transformation (Jia et al. 2024). These approaches can restore blood flow but fail to address secondary damage from inflammation, oxidative stress and ultimately cell death. As a result, neural recovery is limited, and lasting deficits often persist. Consequently, there is an urgent need to develop novel therapeutic strategies that promote neuroprotection, enhance brain repair, and reduce secondary injury after ischemic stroke (Gao et al. 2024).

Extracellular vesicles (EVs) have emerged as promising therapeutic agents in regenerative medicine due to their capacity to deliver bioactive cargo—such as proteins, non‐coding RNAs, mRNAs, and lipids—to recipient cells (Welsh et al. 2024). Acting as natural mediators of intercellular communication, EVs play a crucial role in modulating key physiological and pathological processes, including neuroinflammation, neurogenesis, and angiogenesis. Recent studies have highlighted the potential of EV‐based therapies as a minimally invasive, cell‐free alternative for stroke treatment, offering advantages over traditional cell transplantation approaches by avoiding issues related to cell survival, immunogenicity, and ethical concerns (Kumar et al. 2024; Andjus et al. 2020).

This review explores recent advancements in the therapeutic applications of EVs for ischemic stroke, detailing their mechanisms of action, preclinical and clinical findings, and potential challenges in translation to clinical practice.

### 1.1 Ischemic Stroke: Definition, Epidemiology, and Pathophysiology

The brain serves as the central command system for virtually all bodily functions, and its high metabolic demand requires a continuous and substantial supply of oxygen and glucose. These substrates are essential for sustaining aerobic metabolism, particularly to maintain neuronal activity and action potential propagation. Under physiological conditions, cerebral blood flow averages approximately 50 mL/100 g/min (Feigin et al. 2024). Disruptions in this finely regulated homeostasis—such as reductions in cerebral perfusion—can rapidly lead to profound alterations in the brain microenvironment, impairing neuronal function and viability.

**FIGURE 1:**
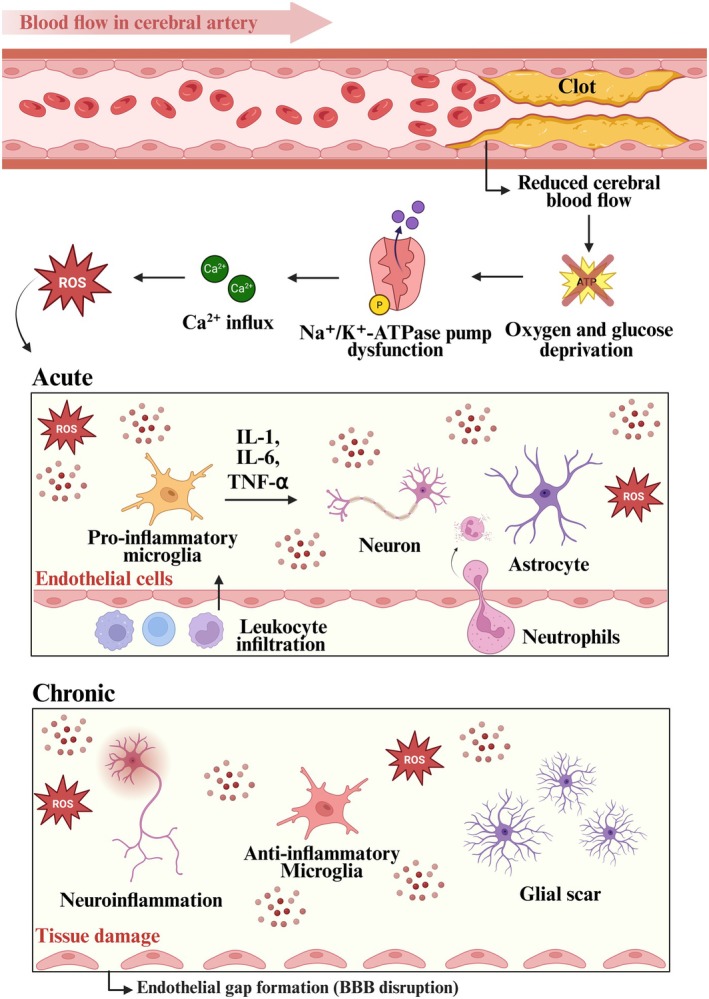
Pathophysiology of ischemic stroke. Schematics showing hemodynamic failure (Oxygen–glucose deprivation), ionic/oxidative stress (Na^+^/K^+^‐ATPase, Ca^2+^, Reactive oxygen species), and phase‐specific responses: Acute inflammation/blood–brain barrier disruption and chronic neuroinflammation with glial scarring.

Stroke is a neurological condition caused by the partial or complete interruption of blood supply to a region of the brain and is broadly classified into two main types: ischemic, resulting from vascular occlusion, and hemorrhagic (Salaudeen et al. 2024). Ischemic stroke represents approximately 87% of all cases (Martin et al. 2025). Vascular occlusion may be caused by embolism, in which a clot originating elsewhere in the body travels to the brain and blocks cerebral blood flow, thereby depriving brain tissue of oxygen and essential nutrients (Majumder 2024). Alternatively, atherosclerosis may contribute to ischemic stroke primarily through plaque rupture and subsequent thrombus formation, or via artery‐to‐artery embolism from unstable atheromatous lesions (Zhao et al. 2022) (Figure 1).

Acute cerebral ischemia creates two zones of tissue injury. The ischemic core represents irreversibly damaged tissue, with cerebral blood flow (CBF) below 10–12 mL/100 g/min, leading to energy failure, ion gradient collapse, calcium overload, enzyme activation, and necrotic cell death. Surrounding the core is the ischemic penumbra, where CBF is reduced to ~10–20 mL/100 g/min. Neurons in this region are electrically silent but structurally intact, making the tissue potentially salvageable if reperfusion occurs promptly. If ischemia persists, the penumbra gradually evolves into the core infarct, a process influenced by hypoperfusion severity, collateral circulation, and the systemic metabolic environment (Ermine et al. 2021; Hilkens et al. 2024).

Ischemia triggers a complex cascade of metabolic disturbances, beginning with ATP depletion and dysfunction of the Na^+^/K^+^‐ATPase pump. This leads to intracellular sodium accumulation and subsequent membrane depolarization (Feske 2021). As a consequence, calcium influx is intensified, provoking excessive glutamate release and overactivation of NMDA receptors, which in turn promotes the generation of reactive oxygen species (ROS). Together, these events contribute to excitotoxicity and progressive neuronal death (Salaudeen et al. 2024) (Figure 1).

Brain regions most vulnerable to ischemia include hippocampal CA1, striatum, neocortical layers III–VI, watershed zones, and Purkinje cells. Glutamatergic neurons are highly sensitive to mitochondrial failure and calcium overload. Endothelial cells, pericytes, and oligodendrocytes contribute to BBB disruption and myelin damage. Astrocytes shift to reactive states with both protective and harmful effects, while microglia rapidly activate, playing dual roles in repair or neuroinflammation (Brookshier and Lyden 2025; Rajput et al. 2024; Liang et al. 2023).

While reperfusion is critical for tissue salvage, it may paradoxically aggravate neural injury. The sudden reintroduction of oxygen and glucose can amplify oxidative stress and mitochondrial dysfunction, further exacerbating damage through mitochondrial depolarization and ROS overproduction (Salaudeen et al. 2024). Mitochondria play a central role in ischemic neuronal death by triggering the opening of the mitochondrial permeability transition pore (MPTP), releasing cytochrome c, and activating caspase‐dependent apoptotic pathways (Peng et al. 2022).

Inflammation is another fundamental component of stroke pathophysiology. Hemodynamic changes and shear stress activate endothelial cells, resulting in the release of pro‐inflammatory cytokines (e.g., IL‐1, IL‐6, IL‐10, TNF‐α) and the upregulation of adhesion molecules such as ICAM‐1 and VCAM‐1, which contribute to blood–brain barrier (BBB) disruption and leukocyte infiltration (Majumder 2024) (Figure 1).

The brain microenvironment concerning cerebral perfusion consists of the neurovascular unit (NVU), a highly integrated network of neurons, endothelial cells, astrocytes, pericytes, microglia, oligodendrocytes, and the extracellular matrix. Together, these components maintain blood–brain barrier (BBB) integrity, neurovascular coupling, cerebral metabolic homeostasis, and the microenvironment necessary for normal brain function (Wang et al. 2025; Alkayed and Cipolla 2023). Under physiological conditions, neurons generate and propagate electrical activity; astrocytes regulate cerebral blood flow and clear excess glutamate (Mishra et al. 2024); endothelial cells and pericytes support BBB tight junctions and vascular tone (Alkayed and Cipolla 2023; Benarroch 2023); and microglia and oligodendrocytes contribute to immune surveillance and myelin maintenance (Alkayed and Cipolla 2023; Wang et al. 2023).

During ischemia, each cell type exhibits distinct pathological responses: neurons rapidly undergo excitotoxicity and oxidative stress–induced cell death; astrocytes lose their ability to regulate vascular tone and neurotransmitter homeostasis (Mishra et al. 2024); endothelial cells and pericytes promote BBB breakdown through tight junction disruption and matrix metalloproteinase activity (Alkayed and Cipolla 2023; Benarroch 2023); and microglia mount an exaggerated inflammatory response in the peri‐infarct area while glia–vascular cross‐talk shapes tissue (Alkayed and Cipolla 2023; Wang et al. 2023). These dynamic and interconnected responses highlight the central role of the NVU in the evolution of ischemic injury and in shaping the brain's capacity for repair.

Current therapeutic strategies for ischemic stroke primarily aim to restore cerebral perfusion to the affected regions. While these interventions—such as thrombolysis and thrombectomy—are effective in re‐establishing blood flow, they do not directly target the secondary cascades of neuroinflammation and oxidative stress that significantly contribute to delayed neuronal injury and long‐term functional impairment. Consequently, the restoration of the neural microenvironment remains incomplete, often resulting in persistent neurological deficits. In this context, EVs have garnered increasing attention as a novel therapeutic approach due to their capacity to modulate inflammatory responses, stimulate angiogenesis, and support neurogenesis, thereby offering a multifaceted strategy to enhance brain repair and functional recovery.

### 1.2 EVs as Emerging Therapeutics for Ischemic Stroke

EVs are lipid bilayer‐enclosed particles naturally secreted by cells, tissues, and biological fluids. They serve as essential mediators of intercellular communication by transporting bioactive molecules such as proteins, lipids, mRNAs, and non‐coding RNAs (Welsh et al. 2024; Yuan et al. 2020). Due to their biological versatility, EVs have emerged as promising tools in diagnostics, therapeutics, and regenerative medicine.

**FIGURE 2:**
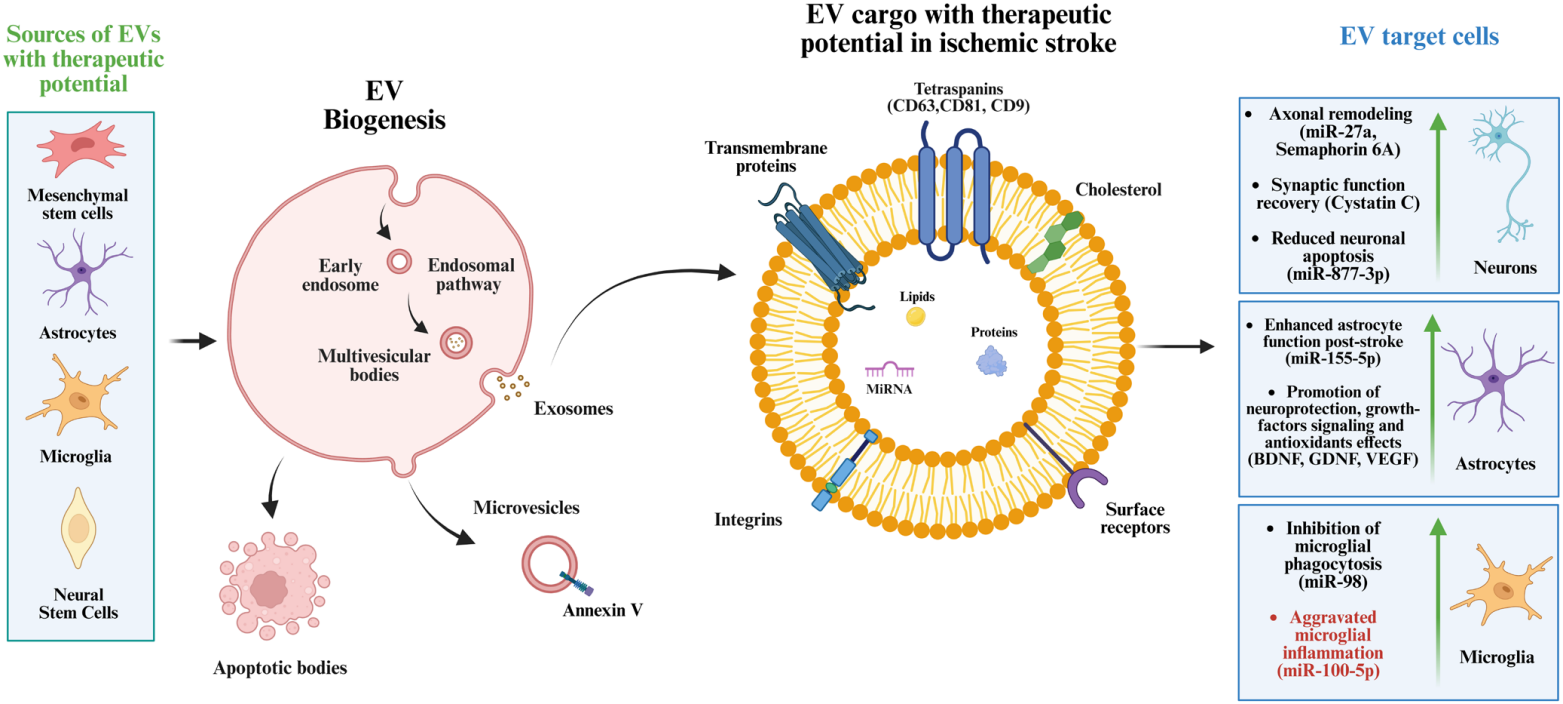
Extracellular vesicles sources, biogenesis, and cargo (tetraspanins/integrins, proteins, lipids, miRNAs). Schematics showing proposed actions in ischemic stroke: neuronal remodeling, astrocyte support, and microglial modulation.

According to the International Society for Extracellular Vesicles (ISEV), EVs can be classified by size—into small (sEVs) and large vesicles—or by their biogenesis into three main types: exosomes (< 200 nm, originating from the endosomal pathway), microvesicles (also known as ectosomes, derived from the plasma membrane and exhibiting a broader size range), and apoptotic bodies (800–5000 nm, released from cells undergoing programmed cell death) (Welsh et al. 2024; Sheta et al. 2023; Meldolesi 2018; Liu et al. 2018; Crescitelli et al. 2013). Exosomes and microvesicles/ectosomes differ in their biogenesis, molecular content, and mechanisms of action, which may directly influence therapeutic effects in the context of ischemic stroke. While exosomes originate from the endosomal pathway and contain proteins associated with multivesicular bodies, specific lipids, and regulatory RNAs, such as miRNAs, microvesicles are released directly by budding from the plasma membrane. Apoptotic bodies originate from the fragmentation of cells undergoing programmed cell death (Jeppesen et al. 2023). In particular, exosomes have attracted significant attention due to their small size, which may enhance their theoretical potential to cross the BBB (Zheng et al. 2019, 2023) (Figure 2).

The molecular cargo of EVs reflects the physiological state of their cells of origin, directly influencing their functional properties. This cargo composition is highly dependent on the cell type and the conditions under which the EVs are secreted or isolated (Welsh et al. 2024) (Figure 2). Among the most studied are EVs derived from mesenchymal stem cells (MSCs), which have demonstrated robust anti‐inflammatory and pro‐regenerative activities. These vesicles can be enriched with anti‐inflammatory cytokines, growth factors, and regulatory miRNAs capable of suppressing microglial and astrocytic activation, thereby mitigating neuroinflammation, carrying a repertoire of bioactive factors that contribute to wound healing, immunomodulation, and angiogenesis (Costa‐Ferro et al. 2024; Xian et al. 2019; Bahram Sangani et al. 2021; Dong et al. 2021; Lai et al. 2010), and have shown efficacy in preclinical models of cardiovascular, pulmonary, and neurological diseases (Yuan et al. 2020).

Beyond their intrinsic therapeutic effects, EVs are being actively explored as natural delivery systems. Their capacity to cross biological barriers, protect encapsulated molecules from enzymatic degradation, and be engineered for targeted delivery enhances their potential for transporting small molecules, RNAs, and therapeutic proteins (Lamptey et al. 2022; Choi et al. 2013; de Jong et al. 2020). This multifunctionality positions EVs as promising tools in the development of next‐generation therapies.

EVs deliver their therapeutic cargo through a combination of targeting, uptake, and controlled release into recipient cells. Their surface ligands, such as tetraspanins (CD9, CD63, CD81), integrins, and adhesion molecules, interact with receptors on target cells, enabling specific binding and internalization via endocytosis, phagocytosis, or direct membrane fusion. Once inside, EVs release bioactive molecules like RNAs, proteins, and lipids, which modulate cellular pathways to drive therapeutic effects (Jeppesen et al. 2023; Jankovičová et al. 2020). Beyond their intrinsic cargo, EVs acquire a biomolecular corona when exposed to biological fluids, composed of adsorbed proteins, lipids, and nucleic acids. This corona dynamically reshapes EV identity by influencing biodistribution, immune recognition, and cellular uptake. In pathological environments such as stroke, the corona can enhance targeting to inflamed endothelium or modulate interactions with immune cells, thereby amplifying or refining their therapeutic impact (Song et al. 2024; Heidarzadeh et al. 2023; Esmaeili et al. 2025).

In the context of ischemic stroke, EV‐based interventions may offer neuroprotective benefits by attenuating inflammation, promoting neuronal survival, and facilitating functional recovery. An important consideration for EV‐based therapies is the route of administration. Intravenous injection remains common in preclinical models, but alternative routes such as intranasal, intrathecal, or localized delivery have shown improved targeting to the ischemic brain, reduced systemic clearance, and enhanced therapeutic efficacy (Pei et al. 2019; Mahdavipour et al. 2020; Zhou et al. 2023; Li et al. 2021).

When compared with synthetic nanoparticles, the unique structure and composition of EVs enable them to serve as natural nanocarriers, along with their physicochemical properties and biological functions. EVs have been reported in preclinical studies to cross the BBB, carry functional biomolecules with immunomodulatory or neuroprotective potential, something synthetic platforms can only mimic with limited success (Zheng et al. 2019; Herrmann et al. 2021; Gurunathan et al. 2021). Being endogenous particles, EVs are considered to have a potentially higher safety profile compared to synthetic particles.

Early preclinical studies provided important groundwork for understanding the potential of EVs in ischemic stroke. Notably, systemic administration of MSC‐derived EVs in a rodent model of MCAO can improve postischemic neurological impairment and induce long‐term neuroprotection associated with enhanced angioneurogenesis (Doeppner et al. 2015). Besides that, another study showed that MSC‐derived EVs can improve functional recovery, increase axonal plasticity and neurite remodeling in the ischemic boundary zone in post‐stroke rats via the transfer of miR‐133b (Xin et al. 2013). Another study showed that astrocyte‐derived EVs could attenuate OGD‐induced neuron death and apoptosis by delivering miR‐92b‐3p, highlighting the contribution of EVs from multiple brain‐resident and peripheral sources (Xu et al. 2019). Collectively, these foundational findings established the rationale for the subsequent surge in EV‐focused research and underscore their promise as a cell‐free therapeutic approach for stroke.

## 2 Methodology

This study is a narrative review of the literature. The PubMed database (https://pubmed.ncbi.nlm.nih.gov/) was searched between January and March 2025. The search strategy employed the descriptive terms “EVs” and “Ischemic Stroke” combined using the Boolean operator “AND” to identify publications addressing both topics simultaneously.

An initial screening of titles and abstracts was performed to identify studies that reported detailed protocols for EV production and discussed their potential applications in clinical translation. Articles meeting these criteria were retrieved and reviewed in full. Only studies published from 2019 to 2025 were considered for this review.

Inclusion criteria were as follows: (1) original experimental studies, (2) published within the last 5 years, and (3) focused on the application of EVs in ischemic stroke research. Only articles published in English were considered. Exclusion criteria included review articles, letters to the editor, and conference abstracts.

Relevant data extracted from the selected studies included journal impact factor, year of publication, experimental methods, key findings, and novel contributions. Some studies are discussed in detail in the Results section, while others are summarized in tables highlighting their main findings and scientific relevance.

## 3 Results

### 3.1 Therapeutic Applications of EVs in Ischemic Stroke

Although numerous studies have shown that cell‐based therapy holds promise for improving functional outcomes following ischemic stroke (Li et al. 2021; Kawabori et al. 2020), several limitations of transferring living cells remain, including risks of tumorigenicity, immune rejection, and challenges related to cell survival and engraftment. In this context, EVs have gained attention as acellular alternatives that can circumvent many of these drawbacks while still exerting neuroprotective, modulating inflammation, and pro‐regenerative effects. Tables 1, 2, 3 summarize studies exploring the therapeutic potential of EVs in ischemic stroke models. Table 1 focuses on EVs derived from ectodermal sources, such as neural stem cells and astrocytes, highlighting their roles in neuroprotection, anti‐inflammation, and neural regeneration. Table 2 presents studies employing EVs from mesodermal origins, including mesenchymal stem cells and endothelial progenitor cells, emphasizing their effects on angiogenesis, immune modulation, and tissue repair. The table shows different sources of EVs and their main functions or molecular mechanisms associated with neuroprotection and modulation of the inflammatory response in experimental models of cerebral ischemia. And Table 3 shows different sources of EVs and their main functions or molecular mechanisms, highlighting the therapeutic potential of these approaches in post‐ischemic neuronal repair.

**TABLE 1:** Extracellular vesicle‐based therapies from ectoderm‐derived sources in ischemic stroke models.

Model	EV source (cell type + condition)	Functional outcome	Route	References
MCAO (rat)	NSC‐derived EVs (Neural stem cells)	Reduced lesion volume, microgliosis, apoptosis; increased neuronal survival	ICV	(Mahdavipour et al. 2020)
OGD (in vitro), MCAO (mice)	Astrocytes‐derived exossomes (Astrocytes)	Increased cell viability and inhibition of neuronal apoptosis. Reduction of inflammation and infarct volume	IV	(Pei et al. 2019)
OGD/R (in vitro)	Neuronal EVs (N2A neuronal cells)	EVs from injured neurons impair UC‐MSC survival; hypoxia preconditioning improves outcome	N/A	(Raffaele et al. 2021)
MCAO (mouse)	Microglia‐derived EVs (activated microglia)	Promoted OPC differentiation, remyelination, and improved functional recovery	IC	(Raffaele et al. 2021)
OGD (in vitro), stroke model (mouse)	Microglia‐derived EVs (hypoxia‐preconditioned microglia)	Promoted angiogenesis and reduced apoptosis via TGF‐β/Smad2/3 pathway	IV	(Zhang, Wei, et al. 2021)
tMCAO (mice)	Extracellular vesicles (EVs) (activated microglia)	Reduced brain atrophy, promoted recovery of sensorimotor and cognitive functions, and enhanced oligodendrogenesis.	IV	(Li et al. 2022)
OGD (in vitro), MCAO (in vivo)	Microglia‐derived EVs (M2‐polarized microglia)	Improved BBB integrity, reduced inflammation and neuronal apoptosis	IV	(Pan et al. 2024)
tMCAO (mouse)	Hypoxic microglia‐derived EVs (human microglia under hypoxia)	Enhanced microvascular density and functional recovery	IP	(Testa et al. 2024)

Abbreviations: EV, extracellular vesicles; IC, stereotactic intracerebral infusion; ICV, intracerebroventricular injection; IP, intraperitoneal injection; IV, intravenous injection; MCAO, middle cerebral artery occlusion; N/A, non applicable; NPC, neural progenitor cell; NSC, neural stem cell; OGD, oxygen–glucose deprivation; OGD/R, oxygen–glucose deprivation/reperfusion; OPC, oligodendrocyte progenitor cell; TGF‐β, transforming growth factor beta; tMCAO, transient middle cerebral artery occlusion; UC‐MSC, mesenchymal stem cells from umbilical cord.

**TABLE 2:** Extracellular vesicle‐based therapies from mesoderm‐derived sources in ischemic stroke models.

Model	EV source (cell type + condition)	Functional outcome	Route	References
OGD (in vitro), MCAO (mice)	BMSC‐derived exossomes (human mesenchymal stem cells from bone marrow)	Promote neuroprotective effects by reducing neuronal injury, decreasing cerebral infarction, and enhancing neuronal survival	IV	(Deng et al. 2019)
OGD (in vitro), MCAO (mice)	MSC‐derived sEVs (human mesenchymal stem cells)	Reduced neurological deficits, infarct volume and cell damage, as well as preserving the integrity of the blood–brain barrier, and attenuated brain inflammation	IV	(Wang et al. 2020)
OGD (in vitro)	BMSC‐derived sEVs (human mesenchymal stem cells from bone marrow)	Prevented OGD/R‐induced neuronal apoptosis, reduced LDH leakage, and an increased Bcl‐2/Bax ratio.	N/A	(Gu et al. 2021)
MCAO (rat)	HUCPVC‐derived EVs (human umbilical cord perivascular cells)	Reduced apoptosis, improved function, increased neuronal density in IBZ	ICV	(Seifali et al. 2020)
OGD (in vitro), MCAO (rat)	iPSC‐derived MSC EVs (human iPSC‐derived MSCs)	Reduce infarct volume, promote angiogenesis and improve neurological deficits	IV	(Xia et al. 2020)
MCAO (in vivo) and OGD/R (in vitro)	ASC‐derived EVs (adipose‐derived mesenchymal stem cells)	Reduced apoptosis and infarct volume by modulating the KDM6B/BMP2/BMF axis	ICV	(Zhang, Liu, et al. 2021)
tMCAO/reperfusion (mouse)	hPMSC‐derived EVs (human placental mesenchymal stem cells)	Preserved cerebral blood flow and reduced infarction	IP	(Barzegar et al. 2021)
tMCAO (rat)	iPSC‐derived EVs + electroacupuncture (human iPSC + EA treatment)	Reduced infarct volume and inflammation; synergistic effect of EA + EVs	IV	(Deng et al. 2022)
Permanent MCAO (rat), OGD (in vitro)	EPC‐derived EVs (EPCs treated with Houshiheisan)	Improved angiogenesis, cortical perfusion, neurological outcomes	IV	(Zhang et al. 2024)

Abbreviations: ASC, adipose‐derived stem cell; BMF, Bcl‐2 modifying factor; BMP2, bone morphogenetic protein 2; BMSC, bone marrow‐derived mesenchymal stem cell; EA, electroacupuncture; EPC, endothelial progenitor cell; EVs, extracellular vesicles; hPMSC, human placenta‐derived mesenchymal stem cell; HUCPVC, human umbilical cord perivascular cell; IBZ, ischemic boundary zone; ICV, intracerebroventricular injection; IP, intraperitoneal injection; IPSCs, induced pluripotent stem cells; IV, intravenous injection; KDM6B, lysine demethylase 6B; LDH, lactate dehydrogenase; MCAO, middle cerebral artery occlusion; MSC, mesenchymal stem cell; N/A, non applicable; OGD, oxygen–glucose deprivation; OGD/R, oxygen–glucose deprivation/reperfusion; tMCAO, transient middle cerebral artery occlusion.

**TABLE 3:** Molecular cargo of extracellular vesicles and mechanisms of action in ischemic stroke models.

Model	EV source (cell type + condition)	Main molecular cargo	Target cell type	Detailed molecular mechanisms	Functional outcome	Route	References
MCAO (mice)	NPC‐derived EVs (neural progenitor cell line)	RGD‐modified EVs (RGD‐EVs)	Ischemic brain endothelial cells	RGD peptide binding to αvβ3 integrin on endothelial cells; Enhanced EV tropism to ischemic area via integrin‐mediated targeting; Reduced inflammatory cytokine production (IL‐1β, TNF‐α)	Reached the lesion area and reduced inflammation after ischemia; Increased EV accumulation in ischemic region; Improved neurological function	IV	(Tian et al. 2021)
tMCAO (mice)	Extracellular vesicles (EVs) (rat cortical neurons)	miR‐98	Microglia	miR‐98 inhibits microglial phagocytosis by targeting CDK5R1/p35 pathway; Prevents phagocytosis of stressed but viable neurons; Downregulates pro‐inflammatory cytokines	Inhibition of microglial phagocytosis, preventing neuronal death; Reduced neuroinflammation	ICV	(Yang et al. 2021)
OGD (in vitro), MCAO (mouse)	Endothelial‐derived EVs (brain endothelial cells)	miR‐155‐5p	Astrocytes	miR‐155‐5p modulates astrocyte function via targeting specific astrocytic genes; Enhances astrocyte‐mediated neuroprotection; Promotes astrocyte proliferation and survival	Enhanced astrocyte function post‐stroke; Improved neuroprotection	IV	(Gao et al. 2023)
MCAO (mouse), OGD (in vitro)	Endothelial EVs (mitochondria‐containing) (brain endothelial cells)	Mitochondria, HSP27	Neurons, Endothelial cells	Mitochondrial transfer restores cellular bioenergetics; HSP27 protects against oxidative stress and apoptosis via inhibition of caspase‐3; Enhanced ATP production and mitochondrial membrane potential	Reduced infarct size, enhanced ATP production, improved mitochondrial function; Decreased apoptosis	IV	(Dave et al. 2023)
Stroke model with Ara‐C (mice)	Neural progenitor cell EVs (neural progenitor cells [NPCs])	Proteomic cargo (neuroprotective proteins)	Neurons, Neural progenitor cells	EVs contain neuroprotective proteins that promote neuronal survival and differentiation; Effect persists under inhibited neurogenesis; Contains growth factors and anti‐apoptotic molecules	Promoted neurological recovery; effect visible under inhibited neurogenesis; Enhanced neuronal survival	ICV	(Campero‐Romero et al. 2023)
OGD (in vitro), MCAO (mice)	Extracellular vesicles (sEVs) (microglia cell line)	RVG29 peptide + NR2B9 (sEVs‐COCKTAIL)	Neurons, microglia	RVG29 targets acetylcholine receptors for brain delivery; NR2B9 inhibits NMDA receptor‐mediated excitotoxicity; Bio‐orthogonal click chemistry for surface modification; Enhanced brain targeting and retention	Reduced reactive oxygen species and cell apoptosis; Brain targeting and increased half‐life of sEVs; Improved neuroprotection	IV	(Haroon et al. 2023)
MCAO (rat), OGD (in vitro)	Endothelial‐derived sEVs (cerebral endothelial cells)	miR‐27a, Semaphorin 6A	Neurons	miR‐27a promotes axonal growth via targeting specific axonal guidance molecules; Semaphorin 6A enhances axonal remodeling; Promotes neurite outgrowth and synaptic formation	Promoted axonal remodeling and neurological recovery; Enhanced synaptic connectivity	IV	(Zhang et al. 2025)
MCAO (mouse)	Neuron‐derived EVs (post‐stroke activated neurons)	miR‐100‐5p	Microglia, Neurons	miR‐100‐5p activates microglial inflammatory response; Promotes pro‐inflammatory cytokine release (IL‐6, TNF‐α); Enhances microglial activation and neuroinflammation	Aggravated microglial inflammation and neuronal dysfunction; Increased neuroinflammation	ICV	(Xin et al. 2024)
Photothrombotic stroke model (mouse)	EVs with circRNA (brain‐targeting EVs loaded with circSCMH1)	circSCMH1	Neurons, Mitochondria	circSCMH1 inhibits KMO expression; Promotes mitochondrial fusion via MFN1/2; Inhibits mitophagy via PINK1/Parkin pathway; Reduces kynurenine pathway activation; Enhances mitochondrial biogenesis	Enhanced mitochondrial fusion, inhibited mitophagy, improved brain repair; Reduced oxidative stress	IV	(Wang et al. 2024)
tMCAO (in vivo) and OGD (in vitro)	Brain‐derived EVs (mouse brain‐derived EVs loaded with cystatin C)	Cystatin C	Neurons	Cystatin C inhibits cathepsin B activity; Protects synaptic proteins from degradation; Maintains synaptic integrity; Transient endogenous protection mechanism in penumbra; Preserves synaptic structures	Promoted synaptic preservation and functional recovery; Enhanced synaptic density	ICV	(Gui et al. 2024)
tMCAO (mice)	hMSC‐derived EVs (human mesenchymal stem cells)	BDNF‐enriched EVs (BDNF‐sEVs)	Neurons, Peri‐infarct region	BDNF binds to TrkB receptors; Activates PI3K/Akt and MAPK/ERK pathways; Promotes neuronal survival and synaptic plasticity; Enhanced targeting to peri‐infarct region	Targeted the peri‐infarct region; BDNF enrichment enhanced therapeutic efficacy; Improved functional recovery	IN	(Zhou et al. 2023)
MCAO (rat), OGD (in vitro)	DPSC‐derived EVs (dental pulp stem cells)	miR‐877‐3p	Neurons	miR‐877‐3p targets pro‐apoptotic genes; Inhibits caspase‐3 activation; Promotes anti‐apoptotic Bcl‐2 expression; Reduces Bax/Bcl‐2 ratio; Protects against OGD‐induced apoptosis	Reduced neuronal apoptosis and improved early neurological recovery; Enhanced cell survival	IV	(Miao et al. 2024)

Abbreviations: Ara‐C, cytosine β‐D‐arabinofuranoside; BDNF, brain‐derived neurotrophic factor; circSCMH1, circular RNA SCMH1; DPSC, dental pulp stem cell; EVs, extracellular vesicles; hMSC, human mesenchymal stem cell; ICV, intracerebroventricular injection; IN, intranasal injection; IP, intraperitoneal injection; IV, intravenous injection; KMO, Kynurenine 3‐monooxygenase; MCAO, Middle Cerebral Artery Occlusion; miR, microRNA; NPC, neural progenitor cell; OGD, oxygen–glucose deprivation; RGD, Arginine‐Glycine‐Aspartic acid peptide; RVG29, Rabies Virus Glycoprotein 29 peptide; sEVs, small extracellular vesicles; tMCAO, transient middle cerebral artery occlusion; TrkB, tropomyosin receptor kinase B.

The intravenous (IV) administration, commonly via tail vein injection, is the most prevalent route for delivering EVs in ischemic stroke models (Pei et al. 2019; Tian et al. 2021; Zhang et al. 2024). It allows controlled dosing and repeat administration, but unfortunately a significant portion of EVs is uptaken by peripheral organs such as the liver, spleen, and kidneys. Another option is the local administration, such as intracerebroventricular (ICV), which provides precise targeting to specific injury sites and has demonstrated a potent reduction in infarct size and neuronal apoptosis (Mahdavipour et al. 2020; Raffaele et al. 2021; Yang et al. 2021). Despite this, it is highly invasive, and is less practical for clinical translation. Alternatively, intranasal (IN) delivery has shown superior brain targeting efficiency in rodent models (Zhou et al. 2023). IN‐administered EVs reach ischemic brain regions as early post‐administration and achieve higher retention in brain tissue compared to IV delivery, but also a fair amount of EVs can accumulate in the peripheral organs (Li et al. 2021).

### 3.2 MSC‐Derived EVs

MSCs are stromal cells with the capacity to self‐renew and also develop multilineage differentiation. MSCs can be isolated from a variety of tissues, such as umbilical cord, bone marrow, adipose tissue, etc. MSCs were first tested as a cellular pharmaceutical in human subjects in 1995 by Hillard Lazarus and have since become the most clinically studied experimental cell therapy platform worldwide. These cells have the advantage of being easily obtained from different sources and are most practical for experimental and possible clinical applications (Ding et al. 2018; Galipeau and Sensébé 2018).

Among non‐neural cell sources, mesenchymal stem cells (MSCs) are the most extensively investigated as a source of EVs for stroke therapy. In vivo, MSCs are considered the functional counterparts of perivascular stromal cells, often referred to as pericyte‐like cells. These perivascular cells reside in close association with blood vessels in multiple tissues, where they contribute to vascular stability, tissue repair, and immune surveillance (Caplan 2017; Crisan et al. 2008). This perivascular niche explains their broad tissue distribution and their ability to rapidly respond to injury by migrating to sites of damage and modulating the local microenvironment. These features—multipotency, immunoregulatory capabilities, trophic factor secretion, and accessibility from various tissues—make MSCs an extensively studied and clinically relevant source for EV isolation. Besides that, these cells need continuous donor screening and introduce inherent variability, as they are intrinsically heterogeneous.

Mesenchymal stem cell‐derived MSC‐EVs have demonstrated considerable therapeutic potential in preclinical models of ischemic stroke; MSC‐EVs also deliver pro‐regenerative factors such as VEGF, BDNF, and pro‐neurogenic miRNAs, which support angiogenesis, neurogenesis, synaptic plasticity, and axonal repair (Costa‐Ferro et al. 2024; Xiao et al. 2025; Xin et al. 2017; Zhang et al. 2017; Xue et al. 2021). Exosomes derived from bone marrow MSCs (BMSC‐sEVs) significantly reduced leukocyte infiltration—particularly neutrophils and monocytes/macrophages—and attenuated microglial activation in the ischemic brain. These effects translated into neuroprotection, evidenced by a decrease in TUNEL‐positive cells and lower ICAM‐1 expression levels (Wang et al. 2020).

Moreover, EVs derived from MSCs generated from induced pluripotent stem cells (iPSCs) enhanced angiogenesis by promoting endothelial cell migration and tube formation. This angiogenic effect was associated with inhibition of endothelial autophagy and resulted in sustained neurological recovery over time (Xia et al. 2020).

Several mechanistic studies have elucidated signaling pathways modulated by MSC‐EVs. One study demonstrated that BMSC‐sEVs attenuate oxidative stress by reducing reactive oxygen species (ROS) levels and upregulating antioxidant enzymes such as superoxide dismutase (SOD) and glutathione peroxidase (GPx) (Gu et al. 2021). These vesicles were also shown to influence the Ca^2+^/CaMK II signaling pathway, a crucial modulator of intracellular calcium dynamics following ischemic injury.

In a bioengineering approach, MSCs were genetically modified to overexpress brain‐derived neurotrophic factor (BDNF), resulting in the production of EVs with enhanced neuroprotective efficacy. These EVs activated the BDNF/TrkB pathway in the ischemic brain, promoting behavioral recovery and neural repair (Zhou et al. 2023).

### 3.3 Microglia‐Derived EVs

Recent investigations have revealed that EVs released from microglia under ischemia‐mimicking conditions exhibit strong therapeutic effects. Microglia‐derived EVs hold strong therapeutic potential because of their CNS‐specific roles as resident immune cells, carrying neurotrophic factors, inflammatory mediators, and microRNAs that modulate neuroinflammation, synaptic remodeling, and neuronal survival. By shaping the microenvironment, microglia support angiogenesis, axonal growth, and cerebral repair through factors such as VEGF, BDNF, IGF‐1, and MMP‐9 (Testa et al. 2024; Zhang, Wei, et al. 2021; Pan et al. 2024). Unlike MSC‐EVs, which are rich in anti‐inflammatory and pro‐regenerative cargo but less brain‐specific, microglia‐EVs provide intrinsic CNS targeting and context‐dependent signaling, making them particularly relevant for neurological diseases (Yan et al. 2024).

EVs isolated from microglia preconditioned with oxygen–glucose deprivation (OGD) were found to be enriched in TGF‐β1, a cytokine associated with the anti‐inflammatory microglial phenotype. These vesicles were capable of crossing the blood–brain barrier and exerted neuroprotective effects by activating the Smad2/3 signaling pathway, leading to enhanced angiogenesis and improved neurological function. These effects were abolished by the inhibition of TGF‐β1 signaling (Zhang, Wei, et al. 2021).

Complementarily, EVs derived from microglia exposed to hypoxia‐reperfusion conditions were shown to regulate both pro‐inflammatory and anti‐inflammatory gene expression programs and actively promote angiogenesis. These findings further reinforce the critical role of microglia‐derived EVs in orchestrating immune and vascular responses following ischemic injury (Testa et al. 2024).

### 3.4 Engineered EVs and Targeted Delivery

EV engineering is a strategy that offers several therapeutic advantages. For example, by modifying their surface with ligands, peptides, or antibodies, it is possible to ensure specific targeting to any tissue of interest, which allows for greater selectivity and reduces off‐target effects. Advances in EV engineering have enabled the development of targeted vesicle‐based therapies for stroke. In addition, engineering strategies, including ligand addition, membrane protein modification, and preconditioning of producer cells, leverage the strengths of naive EVs to improve delivery efficiency and functional outcomes (Murphy et al. 2019; Sun et al. 2023; Liu and Su 2019). For instance, EVs conjugated with Rabies virus glycoprotein 29 (RVG29)—which targets nicotinic acetylcholine receptors—were engineered to carry the neuroprotective peptide NR2B9c. These RVG‐functionalized EVs (RVG‐sEVs) showed prolonged retention in the ischemic brain and efficient delivery of therapeutic cargo to the lesion site. Their neuroprotective activity was associated with increased expression of Bcl‐2 and p38, highlighting their anti‐apoptotic and anti‐oxidative roles (Haroon et al. 2023).

Another study utilized RVG‐targeted EVs to deliver circSCMH1, a circular RNA implicated in neurorepair. The RVG‐circSCMH1‐EVs enhanced mitochondrial fusion and suppressed mitophagy by downregulating kynurenine 3‐monooxygenase (KMO), thereby promoting neuronal survival and recovery (Wang et al. 2024).

In parallel, endothelial cell‐derived EVs enriched with functional mitochondria were combined with heat shock protein 27 (HSP27). This combination improved ATP production and tight junction integrity in brain endothelial cells, contributing to reduced infarct volume and preservation of blood–brain barrier function (Dave et al. 2023).

In another approach, endothelial progenitor cell (EPC)‐derived EVs were bioengineered using Houshiheisan (HSHS), a traditional Chinese formulation known for neurovascular benefits. The resulting EVsHSHS exhibited enhanced therapeutic efficacy by improving cortical perfusion, reducing infarct size, and increasing microvessel density (Zhang et al. 2024).

### 3.5 EVs From Other Neural Cell Types

EVs secreted by other neural cells have also demonstrated therapeutic effects in stroke models. Astrocyte‐derived EVs were shown to enhance neuronal viability, suppress apoptosis, and reduce the production of inflammatory cytokines (Pei et al. 2019). Neuron‐derived EVs were found to modulate microglial activation and prevent phagocytosis of viable neurons, contributing to a neuroprotective environment during recovery (Yang et al. 2021). Synaptosome‐derived EVs loaded with Cystatin C were capable of rescuing synaptic function in the ischemic penumbra, supporting the concept that EVs can preserve circuit integrity after stroke (Gui et al. 2024). Neural progenitor cell (NPC)‐derived EVs have also shown promise. One study reported that NPC‐EVs reduced apoptosis and microgliosis, while improving neurological outcomes following middle cerebral artery occlusion (MCAO) (Mahdavipour et al. 2020). Additional investigations demonstrated that NPC‐EVs could localize to injury sites, reduce ischemia‐induced inflammation (Tian et al. 2021), and stimulate endogenous NPC proliferation (Campero‐Romero et al. 2023).

Other stem cell sources, including human umbilical cord perivascular cells (HUCPVCs), placental MSCs, adipose‐derived stem cells (ASCs), dental pulp stem cells, and iPSC‐derived MSCs, have also yielded EVs with neuroprotective activity. These vesicles carry a diverse repertoire of microRNAs, such as miR‐138‐5p, miR‐22‐3p, and miR‐877‐3p, which mediate anti‐apoptotic, angiogenic, and anti‐inflammatory effects. Collectively, these EVs reduced infarct size, preserved blood–brain barrier integrity, and promoted neuronal survival in various stroke models (Seifali et al. 2020; Barzegar et al. 2021; Miao et al. 2024; Deng et al. 2022).

## 4 Challenges and Considerations

Despite their potential, EVs can also exert harmful effects, particularly when derived from cells under pathological conditions. For example, EVs released by ischemic neurons were shown to exacerbate injury by promoting neurotoxicity and stimulating pro‐inflammatory microglial responses (Xin et al. 2024). Similarly, EVs derived from N2A neuronal cells subjected to oxygen–glucose deprivation induced oxidative stress and neuronal apoptosis (Huang et al. 2020).

These observations demonstrate a dualistic nature of EVs: while many support regeneration and repair, others may amplify damage depending on their origin and the cellular context. Careful selection and characterization of EV‐producing cells, along with optimization of production conditions, are therefore essential for the safe and effective application of EVs in clinical settings.

Regarding safety and immunogenicity, preclinical studies consistently indicate that EVs, particularly those derived from MSCs or other non‐immunogenic sources, exhibit low immunogenicity and are generally well tolerated, even after repeated administration (Doeppner et al. 2015; Kordelas et al. 2014). However, risks related to allogeneic EVs, such as unintended pro‐tumorigenic effects, remain important to monitor in EV studies. The therapeutic efficacy of EVs can vary considerably depending on the parental cell type, donor characteristics, culture conditions, and EV isolation method. Such heterogeneity may account for discrepancies observed across studies and underscores the need for standardized manufacturing and characterization protocols (Welsh et al. 2024; Witwer et al. 2019).

The clinical use of EVs requires overcoming challenges related to large‐scale, Good Manufacturing Practice (GMP)‐compliant EV production, stability during storage, and robust potency assays. Regulatory frameworks for EV‐based therapeutics are still evolving, and clear guidelines on product definition and safety testing will be critical for keeping advancing EVs as a stroke therapy. Besides that, a disease/patient‐specific combination strategy should be explored by small‐scale experiments before manufacturing (Song et al. 2022; Lu et al. 2017).

Despite the encouraging progress in preclinical studies, several challenges remain in the clinical translation of EV‐based therapies for ischemic stroke. One of the major hurdles lies in the isolation and large‐scale production of EVs with high purity and yield, both of which are essential for experimental reproducibility and future commercialization. The inherent heterogeneity of EV populations and their diverse cellular sources necessitate a variety of isolation strategies. However, these techniques often require specialized equipment, orthogonal purification methods, and rigorous characterization protocols to ensure batch‐to‐batch consistency and reliability (Welsh et al. 2024).

A common trade‐off in EV isolation is between purity and yield. For example, precipitation‐based methods offer high recovery rates but often co‐isolate contaminants such as lipoproteins, protein aggregates, and apoptotic bodies, reducing sample purity (Karttunen et al. 2019). Conversely, high‐specificity approaches such as size‐exclusion chromatography or immunoprecipitation—targeting specific surface markers—can achieve greater purity but typically result in lower EV yields (Welsh et al. 2024; Shtam et al. 2020). Therefore, selecting the appropriate method depends on the intended downstream application and may require combinatorial strategies.

Another critical challenge is the variability of EV cargo, even among batches derived from the same cell source. As the therapeutic potential of EVs largely depends on their molecular content—proteins, lipids, and RNAs—this heterogeneity can undermine consistency and efficacy. To mitigate this, it is essential to standardize culture and production protocols and to perform in‐depth characterization using omics tools such as proteomics and RNA sequencing (Welsh et al. 2024; Costa‐Ferro et al. 2024).

The presence of markers such as CD73, CD90, and CD105, along with elevated levels of miR‐21, miR‐146a, and miR‐126 within MSC‐EVs, is frequently used to infer their origin. However, no single molecular cargo is absolutely unique to MSCs. What truly distinguishes MSC‐EVs is their combined and functional profile, particularly their ability to orchestrate immune modulation across multiple cell types and support tissue repair. This multifaceted action—suppressing excessive inflammation, enhancing angiogenesis, and promoting neural survival—underpins their prominence as a therapeutic candidate for neurological disorders.

Moreover, ensuring targeted delivery of EVs remains a key concern. In preclinical and clinical settings, EVs do not always reach the desired tissue or exert effects at the intended site of injury. To address this, several engineering approaches have been developed, including the incorporation of recombinant fusion proteins into the EV membrane to enhance tissue tropism. For instance, the use of RVG peptides has enabled brain‐targeted delivery by interacting with nicotinic acetylcholine receptors (Tian et al. 2021).

In conclusion, while the use of EVs presents a promising therapeutic avenue for ischemic stroke, their successful clinical application depends on overcoming several obstacles. These include the need for robust standardization of production, accurate molecular characterization, improved targeting strategies, and a deeper understanding of potential adverse effects. Nonetheless, their unique features—such as the ability to cross biological barriers, deliver complex therapeutic cargo, and modulate immune and regenerative responses—position EVs as highly attractive candidates for next‐generation stroke therapies.

## Author Contributions

**Elisama Araújo da Silva:** conceptualization, investigation, writing – original draft. **Júlio César Queiroz Figueiredo:** investigation, writing – original draft. **Erik Aranha Rossi:** investigation, writing – review and editing. **Rachel Santana Cunha:** writing – original draft, investigation. **Flávia Santos Sanches:** writing – original draft. **Adne Vitória Rocha de Lima:** writing – original draft. **Erick Correia Loiola:** methodology, writing – review and editing. **Zaquer Suzana Munhoz Costa‐Ferro:** conceptualization, data curation, writing – review and editing. **Bruno Solano de Freitas Souza:** conceptualization, funding acquisition, writing – review and editing.

## Funding

This work was supported by the Brazilian Council for Scientific and Technological Development (CNPq) and D'Or Institute for Research and Education.

## Conflicts of Interest

The authors declare no conflicts of interest.

## Data Availability

The authors have nothing to report.
